# A Playful Experiential Learning System With Educational Robotics

**DOI:** 10.3389/frobt.2020.00033

**Published:** 2020-03-12

**Authors:** Antonella D'Amico, Domenico Guastella, Antonio Chella

**Affiliations:** ^1^Dipartimento di Scienze Psicologiche, Pedagogiche, dell'Esercizio Fisico e della Formazione, Università degli Studi di Palermo, Palermo, Italy; ^2^MetaIntelligenze Onlus, Palermo, Italy; ^3^Centro Interdipartimentale di Tecnologie della Conoscenza, Università degli Studi di Palermo, Palermo, Italy; ^4^RoboticsLab, Dipartimento di Ingegneria, Università degli Studi di Palermo, Palermo, Italy; ^5^Istituto di Calcolo e Reti ad Alte Prestazioni (ICAR), Consiglio Nazionale delle Ricerche, Palermo, Italy

**Keywords:** educational robotics, metacognition, physics, geography, playful-based learning

## Abstract

This article reports on two studies that aimed to evaluate the effective impact of educational robotics in learning concepts related to Physics and Geography. The reported studies involved two courses from an upper secondary school and two courses from a lower secondary school. Upper secondary school classes studied topics of motion physics, and lower secondary school classes explored issues related to geography. In each grade, there was an “experimental group” that carried out their study using robotics and cooperative learning and a “control group” that studied the same concepts without robots. Students in both classes were subjected to tests before and after the robotics laboratory, to check their knowledge in the topics covered. Our initial hypothesis was that classes involving educational robotics and cooperative learning are more effective in improving learning and stimulating the interest and motivation of students. As expected, the results showed that students in the experimental groups had a far better understanding of concepts and higher participation to the activities than students in the control groups.

## Introduction

Technological development in the twenty first century has led to the introduction of new types of technologies in the educational field. One exciting technological innovation is educational robotics (ER), i.e., the application of robotics in an educational context. In this approach, students acquire specific skills (e.g., knowledge of electricity, electronics, robotics) and develop strategic and dynamic capabilities in a playful context that is supposed to increase a learner's motivation and engagement, and facilitate learning. Robotics, indeed, allows the application of the principles of constructivism (Piaget and Inhelder, 1966), constructionism (Papert, 1980, 1993), and embodied cognition (Shapiro, 2010) to learning.

### The Theoretical Background

According to Piaget's principles, cognition develops as an active process in the mental construction of knowledge related to concrete objects in the environment (Piaget and Inhelder, 1966). Papert (1980), starting from Piaget's principles, claimed that learning should not simply be considered the acquisition of behaviors or skills. Instead, it is a subjective process of structuring knowledge, facilitated and enriched by the environmental production of concrete objects defined by the author as “objects to think with” (Papert and Harel, 1991). Papert assigned a high value to concrete thinking, the physical dimension and tangible products of human intelligence (Turkle and Papert, 1992). From his “learning by doing” perspective, ideas are formed and transformed when they are expressed through different tools, when they are used in particular contexts and when individual minds elaborate them. The author deals with the concept of “construction sets”: every mental construction (or robot components, in the case of educational robotics) can be metaphorically associated with parts assembled and built together (Papert, 1980), allowing people “to think with” technological artifacts.

Recently, Raskin (2002) claimed that knowledge is not built through ontogenetically programmed stages of learning, but that it is developed through continuous actions and doing, and the adaptation of the child in the environment with which they interact. In this framework, learning is stimulated by approaches focused on “doing” and on the production of tools that encourage the learner to activate this “constructive” way of learning. Suitable learning contexts should be able to promote: (a) the use of functional strategies to achieve pre-established goals; (b) exposure to different points of view; (c) the involvement of students as an active part of the educational activity; (d) the role of the teacher as a facilitator of the “source of knowledge”; (e) cooperation through the social negotiation of meanings (Vygotsky, 1978); and (f) the use of investigation methodologies as proceeding by trial and error and the activation of problem-solving. Herrington and Kervin (2007) suggest an extension to constructionism by inserting the construct of “authentic activity,” or “poorly defined” activities, requiring students to define the necessary tasks and sub-tasks to complete them. The authors also specify that some kind of “poorly defined” activities may include complex tasks to be investigated over an extended period, which offer students the opportunity to examine the task from different perspectives by using a variety of resources and by providing an opportunity to collaborate and to reflect. We claim that ER satisfies all these requirements for such useful learning contexts.

The importance of educational robotics is also supported by the embodied cognition theoretical approach (Shapiro, 2010), which emphasizes the value of experiential activities in teaching. Embodied cognition is a multidimensional and interdisciplinary construct developed through the contribution of scientific disciplines such as neuroscience, psychology, philosophy, and cognitive sciences. The embodied cognition approach overcomes the debate about the role of the brain/body or the environment in the development of the human mind (Gibbs, 2005). Simply, it considers the body and the environment as an “extension” of the mind. From the perspective of embodied cognition, any kind of human cognition is embodied. In the study of the mind, the role of the body and its interaction with the environment is thus essential. The body ensures coordination between cognition and action, and it facilitates or hinders cognition. Similarly, in ER, learners experience a direct connection between body and mind, and the way the characteristics of the body affect and are affected by the functioning of the mind.

Two main typologies of robots are traditionally employed in the ER field. The first type is classified as a humanoid or zoomorphic robot, as NAO (Shamsuddin et al., 2012) or Pleo (Kim et al., 2013). Robotics construction kits such as LEGO® kits, are a different kind of tool. Humanoid or zoomorphic robots are employed to study human-robot interaction and to improve social skills in children, since they reproduce human or animal-like interactive behavior. Social robots have the advantage of being able to show “human social” characteristics, such as emotions and autonomous language simulation; they can establish/maintain social relationships; they employ natural cues (gaze, gestures, etc.), and learn/develop social competencies (Fong et al., 2003). Social robots are employed in the educational field, for instance, as tutors/teachers or as peers in an educational context (see Belpaeme et al., 2018, for a review). The user does not have the ability of modifying the robot bodies, however, and they are generally expensive, and require advanced expertise in computer programming.

Robotics construction kits, on the contrary, allow the user to build small mobile robots using bricks, sensors, and motors. A simple visual blocks programming language programs the behavior of the robot. These tools stimulate creativity and manipulation. They may be used to perform various activities and to teach various skills in children and adolescents. From our perspective, the use of robotics construction kits is one of the best ways to allow children to work with artificially extended minds.

### The Empirical Evidences

A number of authors have employed robotics construction kits over the past 15 years, for improving cognitive abilities in children with special needs, and for enhancing social and cooperative dimensions and learning in a school context.

In one of our early studies, we documented significant improvements in the academic performance and metacognitive and motivational processes in a student with intellectual disability (Caci and D'Amico, 2005). Similarly, Fridin and Yaakobi (2011) show that robotics can help to improve memory and attention in children with attentional deficit and hyperactivity disorder. More recently, after a robotics lab, we observed improvements in the short-term memory and visual-spatial abilities in a student with visual-spatial difficulties. A child with an intellectual disability and attentional deficit also showed a reduction in behavioral problems (D'Amico and Guastella, 2019). In our 2013 study (Caci et al., 2013a,b), we demonstrated that robotics labs improve the visual-spatial abilities of groups of children with typical development. We also described (D'Amico and Guastella, 2018) how robot construction kits can be adopted in the field of affective computing to support the social and emotional learning of children with typical development or with special needs. In fact, in a study involving a child with autism spectrum disorder, we observed significant changes in social reciprocity and emotional expression, as well as improvements in fine motor skills, verbal and preverbal skills, and a considerable increase in the child's interest in the activity and in-play skills (Guastella et al., 2020).

Many authors have claimed that educational robotics is positively correlated with collaborative learning behaviors, social skills, and the perception of individual and collective self-efficacy (Denis and Hubert, 2001; Kanda et al., 2012). In a school context, the most frequent application of robot construction kits is as a tool for supporting learning in the STEM disciplines (science, technology, engineering, and mathematics). Williams et al. (2007) showed how cooperative learning activities associated with technological tools facilitate the learning of physics in secondary school students, but not in science. Barak and Zadok (2009) aimed to improve learning concepts in Science, Technology, and problem-solving, and showed that the use of a project-based methodology through the use of robotic kits in high school had benefits for an individual's cognitive flexibility, problem-solving and teamwork. Barker and Ansorge (2007) found that an educational robotics course focused on science teaching improved academic performance in a group of students, aged 9 to 11, compared to a control group. Whittier and Robinson (2007) showed that non-English students made significant gains in their conceptual understanding of science concepts after ER activities. Finally, Wei and Hung (2011) showed that ER provides learners with more opportunities for hands-on exercises in mathematics, deepens their perception of the learning contents and that it improved their motivation in the study.

As Toh et al. (2016) claimed, many studies using ER are based on qualitative results, and only one (Whittier and Robinson, 2007) used a quasi-experimental design and provided quantitative measures. Other studies providing quantitative measures are thus needed to confirm the results about learning in STEM disciplines. At the same time, it is crucial to determine whether educational robotics can support learning other than in the STEM disciplines.

Starting from these considerations, we carried out two studies in schools using a quasi-experimental design and employing quantitative tools to measure the impact of educational robotics for fostering the learning of physics and geography. We will first describe the study that involved students from upper secondary school and then the study that involved lower secondary school students, although both studies were carried out independently and at almost the same time by teachers that were been trained in Educational Robotics.

## Study 1

The goal of Study 1 was an evaluation of the application of robot construction kits as tools for supporting the learning of STEM disciplines. In particular, we focused on teaching and learning the concept of physics.

### Method

A quasi-experimental design was used: two classes of the same level were selected. One was assigned to the experimental condition (ER-Physics Lab, described in section The Empirical Evidences) and the second was assigned to the control condition. The class assigned to the control condition attended only one lesson about robotics (focusing on the use of sensors, actuators and processors), but then they attended traditional theoretical lessons about physics, studying the same concepts than students in experimental condition. Both classes shared the same teacher of physics. To test the efficacy of the experimental condition, before and after the whole set of lessons, students in the experimental and control classes completed two questionnaires about physics concepts. We also collected their school marks for physics.

### Participants

A total of 49 students (26 males, 23 females) of about 16 years of age (mean = 16 years, standard deviation = 12 months) participated in the study. They were divided into an experimental and control group. They were from two classes attending the third year of an Italian upper secondary school. One class, including 23 students (11 males, 12 females), was assigned to the experimental condition, and the other class, including 26 students (15 males, 11 females), was assigned to the control condition. There were no students with special educational needs.

Although all the students in experimental condition attended the laboratory, some were excluded from the final analyses since they missed more than two lessons during the laboratory or because they were absent when the pre-test or the post-test was administered (this kind of dropout, unfortunately, is very frequent in studies performed at school). We were thus able to perform the final analyses on a total of 8 students for the experimental group (6 males, 2 females) and 9 students (5 males, 4 females) for the control group.

### The ER-Physics Lab

The class in the experimental condition attended the ER-Physics Lab, which included activities concerning the use of ER for teaching and learning concepts of physics, and particularly the concepts of energy and motion. Lessons were conducted by the teacher, in collaboration with the experimenter from our team, in the role of observer.

Five LEGO Mindstorm EV3 robotics construction kits and five PCs were used in the school's computer lab. The students were divided into five groups so that each group had the opportunity to use one robotic construction kit. The ER activities were carried out in 6 weekly lessons lasting 2 h each, as described below.

#### First Lesson

During the first lesson, the students learned to use the main programming blocks related to the robot motion and functionalities of the sensors. After a brief theoretical introduction, they were free to perform two simple robot motion programs by employing sensors to avoid obstacles, or to stop when a specific condition occurred. At the end of the programming phase, each group showed the program to the other groups, describing the main problems encountered and illustrating the solutions found to solve them.

#### Second Lesson

The second lesson focused on the use of flow programming blocks (loop, switch and wait) and the data operations (variable definition, mathematical operations, and arrays) necessary to code the formulas about motion. The students attended a tutorial and coded a program using these functions. At the end of the programming phase, they showed their results to their classmates.

#### Third Lesson

During the third lesson, after studying formulas for motion calculation, and the respective inverse formulas, each group had to solve a problem with physics using robots. The first step was to estimate the robot's speed. This is a function that is not pre-programmed in EV3 construction kits. To achieve the goal, they employed a meter tool to calculate the length of a path and a stopwatch to measure the time needed by the robot to follow it. Once the students had captured data about space and time, they easily derived the robot speed. The information was then employed to predict the robot's travel time along a straight path or to measure the length of different surfaces, by applying the inverse formulas of the uniform rectilinear motion.

#### Fourth Lesson

At the fourth lesson, the students were asked to implement a program to compute the speed and acceleration of the robot through the radius of wheels and the number of rotations of an engine. The robot was programmed using specific variables (in this case, the time and the number of rotations) and by displaying the results on the computer programming interface or in the small display embodied in the robot. Every time the engine power and the distance traveled were modified, then the program computed and displayed the new speed and acceleration. At the end of the programming phase, each group showed their results to their classmates.

#### Fifth Lesson

During the fifth lesson, the students were asked to calculate the speed of a rotating rod attached to a robot wheel (whose radius was known). The students were also asked to calculate the acceleration of the rod. This request was made in order to activate the students' critical reasoning capabilities, as they had to realize that the required calculation could not be done using the available data only.

#### Sixth Lesson

During the final lesson, students created a program of their choice using the formulas or programming blocks employed in the previous lessons, according to the concepts of physics or programming.

They created exciting programs: Group 1 created a robot that computed its length when dragged on a surface; Group 2 created a robot able to follow a path without impacting against walls or obstacles; Group 3 worked on a variant of the robot that calculates its speed but without using the stopwatch and setting a timer, so that the robot would stop after a period; Group 4 proposed a variant of the robot that calculates distance, was able to display the results of the calculations on the embodied display and to keep the previous measurements in its memory; and finally, Group 5 created a robot able to recognize the color of a spherical object and to carry it to a predetermined position.

### Measures

#### Physics School Marks

The average marks gained by each student in physics before and after the study activity were computed by the teacher. They were based on the student's performance in both written assignments and oral questions and, as usual in Italian schools, ranged from 0 to 10.

#### Questionnaires About Physics and Robotics

In the test and the re-test phase, we employed two parallel questionnaires that covered the topics of physics and robotics and were focused on the periods before and after the lab. Both questionnaires were prepared by the teacher and presented slightly different items, but with the same level of difficulty. They both comprised eight questions: two questions concerned physics problems where the students had to choose the correct formula to solve a problem. The remaining six questions covered topics related to robotics (sensors, actuators, processors, and data collection).

The questionnaires about physics and robotics are to be considered objective measures of learning since they were based on answers given by the students. They were employed to avoid teachers being influenced by the experimental hypotheses when evaluating the students.

Both questionnaires were administered in the classroom, separately for the experimental and control group, in two joint sessions before and after activities.

### Results

A series of 2 × 2 factorial ANOVA using group (experimental, control) × time (test, retest) were performed on data collected during test and retest phases. The analyses were performed on: (a) the average marks in physics; (b) the total score for the questionnaire (QTot); (c) the score related only to questions about physics (QPhys); (d) the score related only to questions about robotics (QRob).

**Graph 1:**
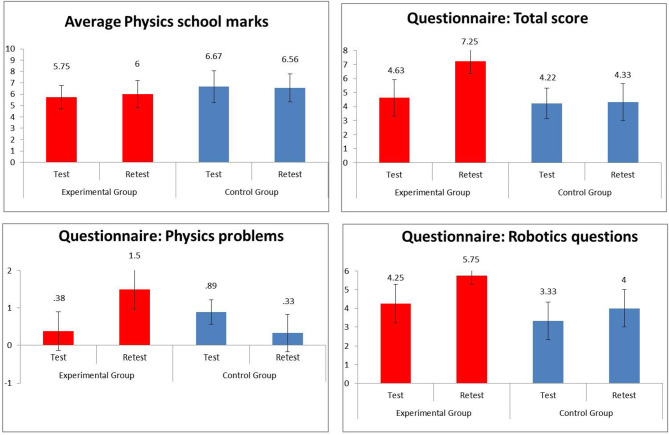
Study 1: Scores of experimental and control group for all variables measured in test and retest phases.

The means and standard deviations of all measures collected in the experimental and control groups before and after the activities and results of statistical analyses are shown in Table 1 and Graph 1.

**Table 1:** Mean and standard deviation of all measures collected in the experimental and control groups and results of statistical analyses.

	**Experimental group**				**Control group**				**ANOVAs**		
	**Before**		**After**		**Before**		**After**		**Group**	**Time**	**Group × Time**
	**M**	**SD**	**M**	**SD**	**M**	**SD**	**M**	**SD**	***F*_**(1, 15)**_**	***F*_**(1, 15)**_**	***F*_**(1, 15)**_**
Average Physics school marks	5.75	1.03	6.00	1.19	6.67	1.41	6.56	1.23	1.69	0.12	0.80
Questionnaire: Total score (QTot)	4.63	1.302	7.25	0.886	4.22	1.093	4.33	1.323	18.87^**^	10.62^*^	8.97^*^
Questionnaire: Physics problems (QPhys)	0.38	0.518	1.50	0.535	0.89	0.33	0.33	0.500	4.21	2.90	25.28^**^
Questionnaire: Robotics questions (QRob)	4.25	1.03	5.75	0.46	3.33	1.00	4.00	1.00	20.53^**^	10.65^*^	1.58

* *p < 0.05*,

** *p < 0.001*.

The results of the Group x Time factorial ANOVA performed on the average marks for physics collected before and after the activities did not reveal a significant main effect for Group [*F*_(1, 15)_ = 1.69, *p* = 0.202, η^2^ = 0.102], Time [*F*_(1, 15)_ = 0.12, *p* = 0.736, η^2^ = 0.008), nor interaction Group × Time [*F*_(1, 15)_ = 0.80, *p* = 0.386, η^2^ = 0.050]. The average marks of the students in the Experimental and Control groups for Physics were thus similar before the start of the activities, and had not substantially changed from test to retest for either the students in the experimental group, nor for those in control groups.

The ANOVA computed on the total score of the questionnaire (Qtot), conversely, showed the significant main effect of Group [*F*_(1, 15)_ = 18.87, *p* = 0.001, η^2^ = 0.557], indicating that, in general, the experimental group obtained better results than the controls in the questionnaire. The significant main effect of Time [*F*_(1, 15)_ = 10.62, *p* = 0.005, η^2^ = 0.415] indicated that all the participants improved their performance in Qtot from the test to the retest. A significant interaction between Group and Time [*F*_(1, 15)_ = 8.97, *p* = 0.009, η^2^ = 0.374] indicated that students in the experimental group improved significantly more than students in the control group.

Significant results were obtained even when only the test scores relative to physics were taken into account (QPhys). The results of the ANOVA indeed showed a main effect of Group very close to statistical significance [*F*_(1, 15)_ = 4.21, *p* = 0.058, η^2^ = 0.219], indicating that, in general, the experimental group obtained better results than the controls in QPhys. The non-significant main effect of Time [*F*_(1, 15)_ = 2.90, *p* = 0.109, η^2^ = 0.162] indicated that when the score of all participants was taken together, there was no improvement from the test to the retest session. A significant interaction between Group and Time [*F*_(1, 15)_ = 25.28, *p* < 0.001, η^2^ = 0.628] indicated that students in the experimental group improved significantly more than students in the control group in answering the questions about physics.

The ANOVA performed on the item of the questionnaire related to questions about robotics only (QRob) demonstrated that there was a significant main effect of Group [*F*_(1, 15)_ = 20.53, *p* < 0.001, η^2^ = 0.578], indicating that students in the experimental group answered better than students in the control group. The main effect of time [*F*_(1, 14)_ = 10.65, *p* = 0.005, η^2^ = 0.415] indicated that both the experimental and control group improved their performance in QRob from test to retest, however, and, unexpectedly, that there was no interaction between Group and Time [*F*_(1, 15)_ = 1.58, *p* = 0.229, η^2^ = 0.095], indicating that there was no significant difference in the improvement of students belonging to the experimental group when compared to students in the control group.

### Discussion

The results of Study 1 demonstrated that the ER-Physics Lab failed to exert a significant improvement in the general knowledge about physics, since we did not find any difference between the experimental and the control group in their average marks for physics, however, it has to be said that none of the students (in the experimental or the control group) showed an improvement in the average physics mark from test to retest, probably because these marks were based on a range of school activities and they generally remain reasonably stable in a narrow period such as that considered in the study.

Remarkably, thanks to the ER-Physics Lab, students in the experimental group obtained a significant improvement compared to students in the control group when answering the questionnaire about physics and robotics administered before and after the activities. An in-depth analysis revealed that the group differences concerned their abilities when solving the two problems with physics. At the same time, there were no significant effects due to the ER-Physics Lab when answering the questions about robotics. This is because the questions about robotics concerned general knowledge about the use of a robot's components, which students in the control group may have acquired during the frontal lesson about robotics. Conversely, solving a problem of physics requires a more in-depth understanding of the physics concepts, and, in this sense, the results demonstrated that the ER-Physics lab allowed students to acquire significant and conceptual learning about the topics in focus during the lab.

Impressive outcomes were derived from the informal observations performed during the lab by the teacher and by the experimenter. They reported active participation by each student group with an evident stimulation of their ability to work in groups, intense collaboration, and a constant comparison among pairs. In particular, during the final lesson, where students were free to create a program of their choice, both teacher and observer reported the emergence of various skills and abilities: some groups focused on solving problems in physics by the different strategies described in sixth lesson; others worked on the programming of the robot (i.e., to follow a track avoiding obstacles or recognizing colors), and others focused on “hardware aspects,” by adapting the robot body according to the task to be performed. Moreover, the teacher perceived an unexpected increase in the performance of the students who were usually less motivated to learn.

## Study 2

Study 2 evaluated the efficacy of ER for teaching and learning concepts of geography about the regions of Italy to students attending the second class of an Italian lower secondary school.

The study has two novel aspects compared to the previous one, and also to the literature in the ER field: the first is that it is focused on geography, a subject that, to our knowledge, has never been considered in studies about educational robotics. The second is that it employs BB8, a small robot by Lego, never previously employed in experimental studies.

### Method

Similarly to study 1, also in study 2 we used a quasi-experimental design: two classes of the same level were selected, and one was assigned to the experimental condition. The second was assigned to a control situation. The performance of both classes in geography was tested before and after the labs.

### Participants

A total of 25 students (11 males, 14 females) of about 12 years of age (mean = 12 years, standard deviation = 12 months) participated in the study. They belonged to two classes attending the first year of a secondary lower level school in Italy. One class, including 12 students (6 males, 6 females), was assigned to the experimental condition, and the other class, including 13 students (5 males, 8 females), was assigned to the control condition. One student with special needs was present in each class; they took part in the same activities performed by classmates.

### The Geography Labs

Differently from Study 1, in Study 2 both classes undertook a laboratory in geography using an innovative method of 3 weekly lessons lasting 2 h each.

In the control condition (GeoLab), the activities described below were carried out in the students' classroom without robots. In the experimental condition (ER-GeoLab) the lessons were carried out using educational robots in the school's computer lab, supervised by an experimenter from our team. The experimenter didn't interact with the students but observed how the lessons developed, intervening only to solve technical problems related to robot functioning. In the ER-GeoLab, five BB8 Sphero toys and five Android Tablets were used. BB8 is composed of two spheres (the head and the body), and it belongs to the Star Wars film saga. It is simple to use and affordable. It may include different behaviors, when programmed using the free iOS and Android compatible apps.

Both laboratories were carried out by two teachers of Italian language, history, and geography. They worked together to avoid differences in the teaching and evaluation methods in the two classes. Before the beginning of the experiment, students in the experimental and control group had the opportunity to play with the robot and to attend a demonstrative session about the ways to assign specific movements to the robot by choosing directions, duration, and speed. The students in both groups were then divided into five groups of 2–3 children each to perform the activities described below.

#### First Lesson

During the first lesson, students in the GeoLab and the ER-GeoLab studied geography by preparing themselves cards for each Italian region, including information about the regional capital and other principal towns, environmental characteristics, economic aspects, and principal artistic and touristic attractions of the regional area. At the end of the activity, however, only the students in the ER-GeoLab had the opportunity to use the robots and to create a program allowing them to move along a blank Italian geography map.

#### Second Lesson

During the second lesson, teachers gave students in the ER-GeoLab target regions in which the robot had to stop. The students thus had a role in programming the robots' movements in order to reach each region and to describe the information about the area, using the “region cards” previously compiled.

Students in the GeoLab carried out a similar activity, but without robots: teachers gave students the target regions, and they had to reach them using a pawn, and then they had to describe the information about the area using the “region cards.”

#### Third Lesson

To consolidate the learning of geography, during the third lesson, the students in the ER-GeoLab performed the same activity as in the second lesson, however, in this case, they could not use the cards, and instead, they had to recollect from memory and repeat the information about each region. Their classmates played a role in verifying the correctness of the given information. Again, students in the GeoLab carried out the same activity but with pawns rather than robots.

### Measures

#### Geography Test

A geography test was given to the students in the experimental and control groups before and after the activities, to determine whether (1) there were differences between groups in geography knowledge before the labs; (2) the two groups showed a different level of geography learning after the ER-GeoLab and the GeoLab. The test consisted of placing the capitals of the Italian regions onto a blank Italian geography map. The students also had to describe the characteristics of the regions (rivers, mountains, primary, secondary, and tertiary sectors). Each student obtained a score from 1 to 10, depending on performance.

As in Study 1, the geography test was used as an objective measure of learning based on the answers given by students, and it was employed so that teachers were not affected by the experimental hypotheses when evaluating the students.

#### School Achievement and Cognitive-Motivational Style

Before the activities, teachers were requested to complete a school achievement grid and cognitive-motivational questionnaire for each student in both groups (as used in Caci and D'Amico, 2005; D'Amico, 2018). This was in order to measure the eventual individual differences in school achievement and cognitive-motivational styles of students in experimental and control groups, which could affect the results of the experiment.

The teachers from the team were requested to report the school marks (from 1, insufficient to 5, excellent) of each student in each of the 13 disciplines taught at school on the school achievement grid. A total school achievement score, ranging from 13 to 65, was then computed for each student.

The cognitive-motivational questionnaire was composed of 18 items that explored the cognitive and metacognitive level (e.g., recognition of their limits, focused attention while performing a task) and motivation (e.g., commitment, curiosity) for each student. For each item, the teachers had to assign a score using a Likert scale from 0 (not at all) to 3 (very much) based on how often they observed each behavioral style in their pupils. A total score, ranging from 0 to 72, was computed for each student.

#### The Pleasantness of the ER-GeoLab Activities

At the end of the activities, only students in the ER-GeoLab group compiled a short questionnaire (see Table 3) aimed at measuring the pleasantness of the activities performed with the BB8 robot.

### Results

**Graph 2:**
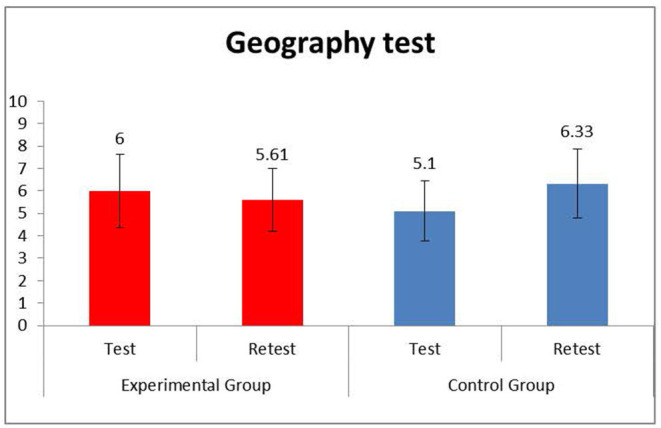
Study 2: Scores of experimental and control group for geography test in test and retest phases.

The mean and standard deviations of all the measures collected in the experimental and control groups and the results of statistical analyses are reported in Table 2 and Graph 2.

**Table 2:** Mean and standard deviation of all measures collected in the experimental and control groups and results of statistical analyses.

	**Experimental group**				**Control group**				**ANOVAs**			***T*-tests**
	**Before**		**After**		**Before**		**After**		**Group**	**Time**	**Group × Time**	
	**M**	**SD**	**M**	**SD**	**M**	**SD**	**M**	**SD**	***F*_**(1, 23)**_**	***F*_**(1, 23)**_**	***F*_**(1, 23)**_**	***t*_**(1, 23)**_**
Geography test	5.1	1.34	6.33	1.55	6	1.63	5.61	1.39	0.010	10.04^*^	39.50^*^	–
School achievement	35.00	9.62	–	–	39.54	15.61	–	–	–	–	–	0.866
Cognitive—motivational questionnaire	32.33	8.99	–	–	35.61	13.60	–	–	–	–	–	0.701

* *p < 0.05*.

To measure differences between the ER-GeoLab and the GeoLab groups in geography knowledge, a 2 × 2 factorial ANOVA using Group (ER-GeoLab, GeoLab) × Time (test, retest) was conducted before and after the labs, on the total geography test scores collected before and after the labs. The results showed that there was no main effect of Group [*F*_(1, 23)_ = 0.01, *p* = 0.922, η^2^ = 0.00], indicating that there was no group difference in the general mean, including the performance of students before and after the labs. There was a significant effect of time [*F*_(1, 23)_ = 10.04, *p* = 0.004, η^2^ = 0.30], indicating that both groups of students significantly increased their competence in geography knowledge after the labs. Finally, there was a significant interaction between Groups and Time [*F*_(1, 23)_ = 39.50, *p* < 0.001, η^2^ = 0.632), indicating that after the lab, students in the ER-GeoLab group improved their knowledge about geography. At the same time, students in the GeoLab group experienced a slight decrease in their performance.

To evaluate whether there were significant differences among groups in school achievement that could have affected these results, a *t*-test was computed on the total scores they had obtained, however, there were no statistically significant group differences [*T*_(1, 23)_ = 0.866, *p* = 0.396], although students in the GeoLab group performed slightly better than students in the ER-GeoLab group (see Table 2). The same result was obtained for the scores of the questionnaire about cognitive and motivational styles: students in the GeoLab group achieved a higher score than students in the ER-GeoLab group (see Table 1), but again the group difference was not statistically significant [*T*_(1, 23)_ = 0.701, *p* = 0.490].

The results for the questionnaire about the pleasantness of the activities performed by students of the ER-GeoLab group with the BB8 robot showed that a high percentage of students evaluated the experience positively (see Table 3).

**Table 3:** Rating by students about the level of the pleasantness of the ER-GeoLab activities.

**Questions:**	**Not at all %**	**A little %**	**Enough %**	**Very much %**
Have the activities been fun?	0	16.66	41.66	41.68
In your opinion, has the use of BB-8 facilitated your learning?	0	0	33.33	66.67
How much did you participate in the activities?	0	0	33.33	66.67

### Discussion

The results of this study demonstrated that the use of ER in a geography lab has significant effects on learning geography. although both experimental and control groups performed similar activities in order to learn information about the Italian regions, only students in the experimental group obtained significant improvements in geography knowledge after the lab. Moreover, it has to be stressed that these results were obtained even though the experimental group reported slightly lower scores in general school achievement and in the questionnaire about cognitive-motivational styles during the school year.

ER stimulated participation and fun in students of the experimental group. They considered the activity facilitated learning, as documented by answers to a questionnaire about the pleasantness of activities.

## General Discussion and Conclusions

In conclusion, the results of the two reported studies showed that the use of robotics at school improved the learning of the students involved in the activities. Although Study 1 and Study 2 are not directly comparable due to their numerous differences, the students belonging to the experimental group in both studies achieved better learning results than the students in the control groups.

In Study 1, even though there were no differences between the groups in their physics marks before the activities, students in the experimental group obtained higher scores than the controls in the school-type problems of physics included in the questionnaire. Thus, as already claimed, they generalized the acquired ability to solve the problem of Physics using robots (learning by doing) to the solution of other types of paper-pencil problems in Physics.

Conversely, and quite surprisingly, students in the experimental group obtained results similar to the controls in the items of the questionnaire that concerned robotics. All considered, it seems that it is enough to address the topic “robots” to improve learning, given its level of interest: indeed, the score on the questions about robotics was high for both groups. This also suggests that if, on the one hand, ER helps students to learn better about traditional subjects such as physics, on the other hand, robotics is such an innovative and attractive topic that students learn basic concepts about it even when a conventional teaching method is used.

In Study 2, we also demonstrated the efficacy of ER for teaching subjects in the humanities and not only in STEM subjects. Even when students in the experimental group had lower scores than the controls in school achievement and cognitive-motivational styles, they improved their knowledge of geography. In contrast, the controls showed a slight decrease in performance from the test to the retest. Moreover, as already claimed, both experimental and control groups realized a non-conventional learning lab, through group activities and playful teaching methodologies, and the only difference between the experimental and control group was the use of robots. We can thus confirm that the results obtained do not depend in general on the use of an innovative educational method, but the use of ER was also considered fun and useful by the students.

Why is robotics so valuable and attractive? There may be many reasons, as we have described in the introduction. From our perspective, the main reason is definitely that educational robotics combines physical and mental experience: according to the constructionist and EC theories, learning and cognitive functioning are affected by the physical and mental experience of interaction with the environment and with the tools it contains. It allows students to learn by doing, to manipulate concepts, and to embody cognition. In ER sessions, students have the opportunity to approach an idea from both an abstract and a concrete point of view. This leads to the creation of different forms of memory, such as semantic memory (i.e., memorizing the role and the function of each component) or the procedural memory (i.e., learning how each part works and how it has to be managed) to create accurate and complete episodic learning.

The second reason, as claimed by many authors, is that robotics may increase motivation for learning in situations that are generally seen by children as passive and not very stimulating. In Study 2, all children participating in ER-GeoLab declared that the activities were fun and useful for learning, and we already know that the so-called “digital native” generation (Prensky, 2001) is undoubtedly more interested in digital and multimedia technologies than previous generations. Children spend more time playing with technological tools than with “non-technological” tools. Beran et al. (2011) and Bullen and Morgan (2011) also demonstrated that this is not only limited to digital natives but also the so-called digital immigrants, people who started to use technologies as adults, and who are sometimes more passionate than children about technologies.

We also stress that the ER is not only a facilitator for students but also teachers. All teachers expressed great interest toward the use of robotics in teaching since it has positive effects on the school climate and may contribute significantly to creating a stimulating learning environment, both for students and teachers. The greater involvement of students in the topics dealt with using the robots makes it easier to keep the class's attention in the long-term and makes the teacher's work more rewarding. During and after the laboratory, the teachers involved in Studies 1 and 2 reported that the activities improved their feeling about the effectiveness of their teaching method, making not only students but also themselves more motivated. They not only consider robotics an excellent tool for supporting teaching, but they also see it as a significant change to the monotony of traditional education. In conclusion, the perception of their work status was improved.

## Limitations and Future Directions

Although the results obtained are encouraging and reinforce the idea that robotics can be considered a valuable teaching tool, there are many limitations in these studies that have to be considered and hopefully overcome in future studies. The first involves the experimental setting chosen for the activities. The school context presents many constraints: (1) it is not possible to realize a very innovative design by assigning single participants randomly to experimental or control conditions. Each class in a school, may participate or not as a whole, mainly due to problems involving the organization of school activities and timetables; (2) activities in groups could be challenging to organize. For instance, the contrasting leadership of two members can result in a group that does not collaborate very well, and the absence of one of the group members may affect the work of the other members; (3) there could be significant differences between two classes, due to the students that belong to them, but also due to the team of teachers, who can have different levels of competence or use different systems of evaluation or didactic methods; (4) In our study we chose classes with the same teachers to reduce these differences in teaching, however, using the same teacher in both experimental and control class, doesn't allow for “blind” testing. The teacher knows which is the experimental and the control class, and they are involved as the first person in the experience. Their increased enthusiasm for the new robot-mediated method may affect the teachers' behavior; (5) more studies involving longer interventions are needed. In particular, it will be necessary in the future to design longer-lasting interventions with different follow-up assessments to clarify whether, behind the initial motivational boost that robotics exerts in digital natives and teachers, robotics-based teaching leaves longer-lasting memory traces in students and allows a more in-depth and meta-cognitive comprehension of the studied topics than traditional methods.

Despite these limitations, it seems to us that educational robotics can have a significant impact on the school.

## Data Availability Statement

The datasets generated for this study are available on request to the corresponding author.

## Ethics Statement

Ethical review and approval was not required for the study on human participants in accordance with the local legislation and institutional requirements. Written informed consent to participate in this study was provided by the participants' legal guardian/next of kin.

## Author Contributions

All authors equally contributed to manuscript preparation and revision, read, and approved the submitted version.

### Conflict of Interest

The authors declare that the research was conducted in the absence of any commercial or financial relationships that could be construed as a potential conflict of interest.
